# Exosomes with FOXP3 from gene-modified dendritic cells ameliorate the development of EAE by regulating the balance of Th/Treg

**DOI:** 10.7150/ijms.72655

**Published:** 2022-07-11

**Authors:** Zhen Jia, Jia Liu, Bin Li, Le Yi, Yanmin Wu, Junna Xing, Liang Wang, Jinli Wang, Li Guo

**Affiliations:** 1 Department of Neurology, The Second Hospital of Hebei Medical University, Shijiazhuang, Hebei, China.; 2 Neurological Laboratory of Hebei Province, Shijiazhuang, Hebei, China.; 3 Department of Neurology, Dongzhimen Hospital, Beijing University of Chinese Medicine, Beijing, China.; 4 Institute for Brain Disorders, Beijing University of Chinese Medicine, Beijing, China.

**Keywords:** Multiple sclerosis, experimental autoimmune encephalomyelitis, exosomes, dendritic cell, FOXP3

## Abstract

**Objective:** To investigate the efficiency and potential mechanisms of exosomes from dendritic cells (DCs) transfected with Forkhead box protein P3 (FOXP3) in the development of experimental autoimmune encephalomyelitis (EAE).

**Method:** Mouse bone marrow-derived immature DCs were loaded with adenovirus carrying FOXP3 gene, and exosomes were generated. Then the exosomes with FOXP3 (FOXP3-EXOs) were co-cultured with CD4+T cell *in vitro* to evaluate their potential on CD4+T cell proliferation and differentiation, and injected into EAE mice to assess their effects on the development of EAE.

**Result:** FOXP3-EXOs were effective to inhibit the CD4^+^T cell proliferation and the production of Interferon gamma (IFN-γ), interleukin (IL)-6, and IL-17, while they promoted the production of IL-10 *in vitro*. Moreover, FOXP3-EXOs treatment significantly decreased the neurological scores, reduced the infiltration of inflammatory cells into the spinal cord, and decreased demyelination in comparison to saline and Con-EXOs treated EAE mice. Moreover, the FOXP3-EXOs treatment resulted in obvious increases in the levels of regulatory T (Treg) cells and IL-10, whereas levels of T helper 1 (Th1) cells, Th17 cells, IFN-γ, IL-6, and IL-17 decreased significantly in the splenocyte culture of EAE mice.

**Conclusion:** The present study preliminarily investigated the effects and potential mechanisms of FOXP3-EXOs in EAE and revealed that the FOXP3-EXOs could inhibit the production of Th1 and Th17 cells and promote the production of Treg cells as well as ameliorate the development of EAE. The neuroprotective effects of FOXP3-EXOs on EAE are likely due to the regulation of Th/Treg balance.

## Introduction

Multiple sclerosis (MS) is an autoimmune related demyelination disease of the central nervous system (CNS) affecting about 2.5 million people worldwide, characterized by recurrent and/or progressive neurological dysfunctions, such as optic neuritis, sensory disturbances, or motor impairments 1, 2. Although the pathogenesis of MS had not been fully elucidated, the interactions between the genetic and environmental factors triggering the abnormal immune responses are generally believed to contribute to the development of this disorder 3, 4. Particularly, accumulating lines of evidences demonstrate that the imbalance of T helper (Th) and regulatory T cell (Treg) plays an essential role in the pathogenesis of MS through disrupting the blood-brain barrier, thereby facilitating the invasion of various inflammatory cells into the CNS, and activating the CNS resident immune cells finally leading to the myelin sheath and axon impairments 5-10. Hence, down-regulation of pro-inflammatory Th1 and Th17 cells, and promotion of Treg responses is expected to be potential strategies protecting against the development of MS. Despite the further understanding of the mechanisms of MS, definitively effective treatments are still lacking. Therefore, it is urgently necessary to explore new clues for therapeutic interventions for MS.

Dendritic cells (DCs) are professional antigen-presenting cells that play a dual role in T cell mediated immunity and tolerance 11. The potential of DCs in immunological modulation depends on their maturation status, in which immature DCs (imDCs) expressing lower levels of costimulatory molecules and histocompatibility complex have a strong ability to take up and process antigens and induce T-cell tolerance, whereas mature DCs (mDCs) induces T-cell immunity 12-14. Forkhead box protein P3 (FOXP3), a member of the Fox family of transcription factors, is a signature surface protein of Treg cells that plays a decisive role in the development and biological function of Treg cells 15, 16. In a previous study, we reported that imDCs transfected with FOXP3: 1) still manifested the imDCs phenotypes; 2) inhibited the proliferation of CD4^+^T cells isolated from the animal model of MS -- Experimental autoimmune encephalomyelitis (EAE) mice; 3) inhibited the production of Th1 and Th17 cell-associated cytokines and enhanced the production of Th2 and Treg cell-associated cytokines, thus reversing the Th/Treg imbalance in the *in vitro* mixed lymphocyte-DCs culture assay 17. In another study, systemic administration of TGF-β1 gene-modified imDCs delayed the development of dextran sulfate sodium induced murine inflammatory bowel disease 18. These demonstrations indicated that through inducing the immune tolerance and regulating the balance of Th/Treg, gene modified DCs may represent a potential treatment strategy for autoimmune diseases. Nevertheless, due to the complexity of live cell vaccines in terms of production and quality control, DC-derived exosomes are receiving increasing attentions in the recent years 19.

Exosome, a small lipid bilayer vesicle could be secreted by all types of cells. They are formed by membrane outgrowth into the inner lumen of the endocytic compartment leading to the formation of multivesicular bodies and once released, exosomes have activities as diverse as remodeling the extracellular matrix and transmitting signals 20, 21. Notably, in addition to being involved in the immunomodulatory processes through the direct cell-cell interaction, DCs may elicit T cell immune responses through the release of biologically active exosomes 19, 22. Accordingly, exosomes from DCs transfected with vIL-10, FasL, or IL-4 gene were reported to inhibit delayed-type hypersensitivity and collagen-induced murine arthritis 23-25. Exosomes with membrane-associated TGF-β1 from imDCs inhibit murine EAE 26. Therefore, exosomes from gene-modified DCs hold promise as a novel strategy for the treatment of autoimmune diseases such as MS through serving as an effective carrier for carrying immunomodulatory molecules.

As mentioned above, our previous study had shed light on the optimal efficiency of FOXP3 gene-modified DCs in inducing immune tolerance and remodeling Th/Treg homeostasis *in vitro*. However, the immunomodulatory effects of FOXP3 gene-modified DCs-derived exosomes *in vitro* and *in vivo* have not been explored. In the present study, we aim to investigate the therapeutic efficiency and potential mechanisms of exosomes from DCs transfected with FOXP3 in EAE.

## Materials and methods

### Mice

The 8-10 weeks old female C57BL/6 mice purchased from the Vital River Laboratories (Beijing, China) were used as donors for the culture of bone marrow-derived DCs (BMDCs) and splenic CD4^+^ T cells. The mice were also served as EAE models and subjected to exosome injections. The experimental protocols were approved by the Animal Care and Use Committee of the second hospital of Hebei medical university (Shijiazhuang, China).

### Generation and transfection of imDCs

#### Isolation and culture of imDCs

The bone marrow-derived mononuclear cells (BMMCs) were prepared from C57BL/6 mice tibia and femur suspensions by depletion of red blood cells and cultured at a density of 2×10^6^ cells/ml in the RPMI 1640 medium supplemented with 10% fetal bovine serum (FBS), 10 ng/mL recombinant mouse granulocyte macrophage colony stimulating factor (rmGM-CSF; PeproTech, Rocky Hill, NJ, USA) and 10 ng/mL rmIL-4 (PeproTech) at 37 °C with 5% CO_2_ for 6 days. On day 6, the imDCs were isolated by negative selection using magnetic beads (CD11c Cell Isolation Kit, Miltenyi biotech, Germany) following the manufacturer indications. In addition, cells were fixed and allowed to adhere to poly-L-lysine coated coverslips before preparation for scanning electron microscopy observation.

#### Construction of Adenovirus-FOXP3 (Ad/FOXP3)

The DNA fragment for mouse FOXP3 in the Ad/FOXP3 was amplified by PCR using specific primers as following: Forward: 5'-CCGGGTACCGGAATTCGCCACCATGCCCAACCCTAGGCC-3' Reverse: 5'-CATGGTGGCGGAATTAGGGCAGGGATTGGAGC-3' synthesized by Shengbo Biotech (Shanghai, China). The shutter vector (pAV-MCMV-GFP-3FLAG) was digested and the linearized vector fragment was recovered. The FOXP3 gene fragment and the linearized vector fragment were homologously recombined and transformed into E.coli receptor cells. Transformants were identified by colony PCR and positive clones were confirm by DNA sequencing. The clones that were sequenced correctly were subjected to plasmid extraction. Subsequently, the shuttle vector or shuttle vector with FOXP3 sequence was co-transfected with pBHGlox E1, 3Cre Ad backbone plasmid into HEK293 cells to generate Ad/Con and Ad/FOXP3. Then, the generated Ad/FOXP3 or Ad/Con was purified and stored at -80 °C until assayed.

#### Transfection of Ad/FOXP3 or Ad/Con to imDCs

Cells were loaded with Ad/FOXP3 or Ad-Con at a multiplicity of infection (MOI=500) and incubated at 37 °C with 5% CO_2_ for 15 minutes. Then, RPMI 1640 medium containing 10 ng/mL rmGM-CSF and 10 ng/mL rmIL-4 were added to culture and incubated at 37 °C with 5% CO_2_ for 24 hours. After that, cells were re-stimulated with rmGM-CSF and rmIL-4 for 48 hours. The efficiency of Ad/FOXP3 transfection was observed under a fluorescence microscope 72 hours after the first stimulation. The imDCs were harvested post-transfection with Ad/FOXP3 or Ad/Con, added with PBS, centrifuged at 1200 rpm for 10 minutes at room temperature, resuspended, and adjusted to a density of 1×10^6^ cells/mL. The supernatants were harvested for the subsequent experiments.

### Exosome isolation and identification

The collected supernatant was centrifuged at 10000 rpm for 30 minutes to remove the cell debris. Then, 250 µl of supernatant was transferred to a fresh tube and incubated with 63 µl ExoQuick-TC Solution as the manufacturers' instruction (SBI Exoquick-TC, USA). After incubation for 12 hours at 4 °C, contents of each tube were centrifuged for 30 minutes at 1500 g, the supernatant was removed by aspiration, and the exosomes pellet was re-suspended in 1000 μL of PBS.

Exosomes pellets were fixed in 4% paraformaldehyde, and then loaded onto electro-microscopy grids coated with formvar carbon, contrasted and embedded in a mixture of uranyl acetate and methylcellulose. Sections were examined with a JEM-1230 electron microscope (EM, JEOL, Japan). The size of exosomes was measured using Particle Metrix ZetaView (Particle Metrix, Germany) following the manufacturer's instructions. Besides, the expressions of exosomes-related proteins and FOXP3 protein were detected by Western blot. Briefly, pre-enriched exosomes samples were lysed in RIPA buffer supplemented with complete Protease Inhibitor Cocktail Tablets (Roche Applied Science, Mannheim, Germany). Equal amounts of proteins were separated by sodium dodecyl sulfate polyacrylamide gel electrophoresis (SDS-PAGE) and transferred onto polyvinylidene fluoride membranes (PVDF, Millipore, USA). Nonspecific binding sites were blocked with 5% skimmed milk in Tris-buffered saline Tween (TBS-T) and then incubated overnight at 4 °C with primary antibodies against CD63 (1:1000, Abcam, USA), HSP70 (1:1000, SANTA CRUZ, USA), and FOXP3(1:1000, Abcam, USA). GADPH (1:2500, Abcam) was used as the internal control. After being washed with TBS-T for three times, the bound antibodies were detected with corresponding secondary antibodies (1:10000, Santa Cruz, USA.) for one hour at room temperature. The PVDF membranes were washing in TBST, then analysis of greyscale values with image J 1.46 software (NIH, Bethesda, USA). The relative levels of target protein to control protein expression were quantified by enhanced chemiluminescence. Results were further assessed using Image J software for density analysis.

### T-cell proliferation assay

Murine splenic CD4^+^ T cells isolated by a CD4^+^ T cell isolation kit (Miltenyi Biotec, Germany) were labeled with CCK-8 (MedChem Express LLC, USA), according to the manufacturer's instructions. Additionally, the labeled CD4^+^ T cells (1 × 10^6^/mL) were stimulated with 1 μL anti-CD3/CD28 coated beads (Thermo-Fisher scientific, USA) with or without different doses of each type of exosome for 3 days. After that, the cells were harvested and analyzed by a spectrophotometer for proliferation.

### EAE induction and treatment

Female wild-type C57BL/6 mice (8-10 weeks of ages, 18-20 g) were randomly into four groups (6 mice in each group). For EAE induction, mice were subcutaneously injected at two sides on the flank with 250μg of MOG_35-55_ (Lysine Bio-system, Xi'an, China) fully emulsified in an equivalent volume of Complete Freund's Adjuvant (CFA, Sigma, St Louis, MO, USA) containing 4mg/ml of heat-killed mycobacterium tuberculosis H37Ra (Difco Laboratories, Detroit, MI, USA). On 0h and 48h after immunization, the mice were injected intraperitoneally with 500 ng pertussis toxin (Alexis, San Diego, USA).

Subsequently, the EAE mice were randomized and treated tail intravenous injection of exosomes (Con-EXOs or FOXP3-EXOs; 10 μg/mouse/injection) or vehicle saline on day 3, 6, 9, 12, 15 postimmunization 26. Another group of control healthy mice were injected with vehicle saline according to the EAE mice injection plan above. The mice were then examined daily for weight and clinical signs. The clinical signs were assessed by using a neurological function scoring system 27. This scale ranges from 0 to 15 and is the sum of the state of the tail and four limbs. For the tail, 0=no signs; 1= half paralyzed tail; 2= fully paralyzed tail. For each of the hind- or forelimbs, each assessed separately: 0=no signs; 1=weak or altered gait; 2= paresis; 3=fully paralyzed limb. Thus, a fully paralyzed quadriplegic animal would attain a score of 14. Mortality equals a score of 15.

### Histology

At 25 days post-immunization, mice in each group (n=6 mice/per group) were subjected to perfusion with 4% (w/v) paraformaldehyde under deep anesthesia (10% chloralhydrate, 0.2ml/mouse, intraperitoneally). Their brains and spinal cords were removed carefully and embedded in paraffin. Three brain sections (5 μm/each, interval of 100 μm) of individual mice were stained with hematoxylin & eosin (HE) and Luxol Fast Blue (LFB) for routine evaluation of inflammatory infiltration and demyelination. The degrees of inflammation and demyelination on three non-serial sections of each mouse were assessed semi-quantitatively in a blinded manner, as described previously. Briefly, for inflammation, the following scale was used: 0, none; 1, a few inflammatory cells; 2, organization of perivascular infiltrates; and 3, increasing severity of perivascular cuffing with extension into the adjacent tissue. For demyelination, the following scale was used: 0, none; 1, rare foci; 2, a few areas of demyelination; and 3, large (confluent) areas of demyelination 28.

### Flow cytometry

To measure T cell differentiation induced by MOG_35-55_, splenocytes were isolated from mice on day 25 post-immunization and stimulated with 100μg/ml of MOG_35-55_ peptide in RPMI1640 medium containing 10% FBS for 48 h. During the last 4h of stimulation, the cells used for Th detection were exposed to 50ng/ml of phorbol-12-myristate-13-acetate (Sigma-Asdrich, USA) and 500ng/ml of ionomycin and 3μg/ml of Brefeldin A (Sigma), and cells for Treg detection were not. After that, cells were stained with FITC-anti-CD4, APC-anti-CD25, fixed, permeabilized, and stained for intracellular cytokines with PE-anti-IFN-γ, APC-anti-IL-4, PE-anti-IL-17A and PE-anti-FOXP3 for Th1, Th2, Th17 and Tregs, respectively (eBioscience, San Diego, CA, USA), followed by flow cytometry analysis on a FACS Calibur flow cytometer (BD Biosciences, San Jose, CA, USA). The cells were gated on living cells and a minimum of 10^4^ cells were assayed. The gating strategy used is described in the following. Firstly, lymphocytes were gated by FSC and SSC. Based on above, CD4+IFN-γ+, CD4+IL-4+, CD4+IL-17+, and CD4+CD25+Foxp3+ lymphocytes were identified as Th1, Th2, Th17, and Treg cell, respectively. Data were analyzed with the Cell-Quest Software (BD Biosciences) in a blinded manner.

### Enzyme linked immunosorbent assay (ELISA)

The concentrations of IFN-γ, IL-4, IL-6, IL-10, and IL-17 (Neobioscience, Shenzhen, China) in the spleen CD4^+^ T cell culture supernatants or serum of mice were determined by commercially available ELISA kit following the manufacturer's instructions.

### Statistical analysis

Data are expressed as mean ± standard error of the mean (SEM). One-way ANOVA analysis followed by least significant difference (LSD) test was applied to assess differences among three or more groups, and Independent-samples T test was used to evaluate differences between two groups. The value of P < 0.05 was considered as a statistical significance. GraphPad Prism7 software was applied for the statistical analysis (MDF Co. Ltd., Tokyo, Japan).

## Results

### imDCs morphology and phenotypes

**Figure 1:**
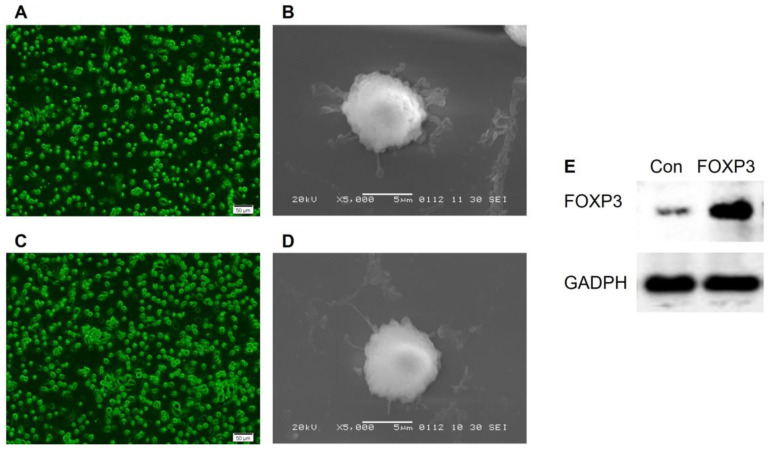
** The morphologic features of imDCs transfected with Ad/Con or Ad/FOXP3. (A)** Fluorescence Microscope (Scale bar = 50 µm) and **(B)** electron microscope (Scale bar = 5 µm) observations of imDCs transfected with Ad/Con. **(C)** Fluorescence Microscope and **(D)** electron microscope observations of imDCs transfected with Ad/FOXP3. **(E)** Western blot analysis of FOXP3 protein expression in the imDCs transfected with Ad/Con or Ad/FOXP3. GADPH was used as the internal control.

BMMCs were cultured in RPMI 1640 medium supplemented with the appropriate cytokine (GM-CSF, IL-4). On day 6 post-culture, the suspension cells were found to increase and the morphology of imDCs gradually appeared. And after CD11c^+^ isolation, the cells manifested a typical morphology of imDCs, such as a circular shape with short cytoplasmic projections. Further, the imDCs post-transfected with Ad/Con (Fig. 1A-B) or Ad/FOXP3 (Fig. 1C-D) still manifested a morphology of imDCs, while a significantly increased expression of FOXP3 were observed in imDCs transfected with Ad/FOXP3 than those transfected with Ad/Con indicating a successful construction of imDCs with FOXP3 over-expression (Fig. 1E).

### Exosome morphology, size, and target protein expression

**Figure 2:**
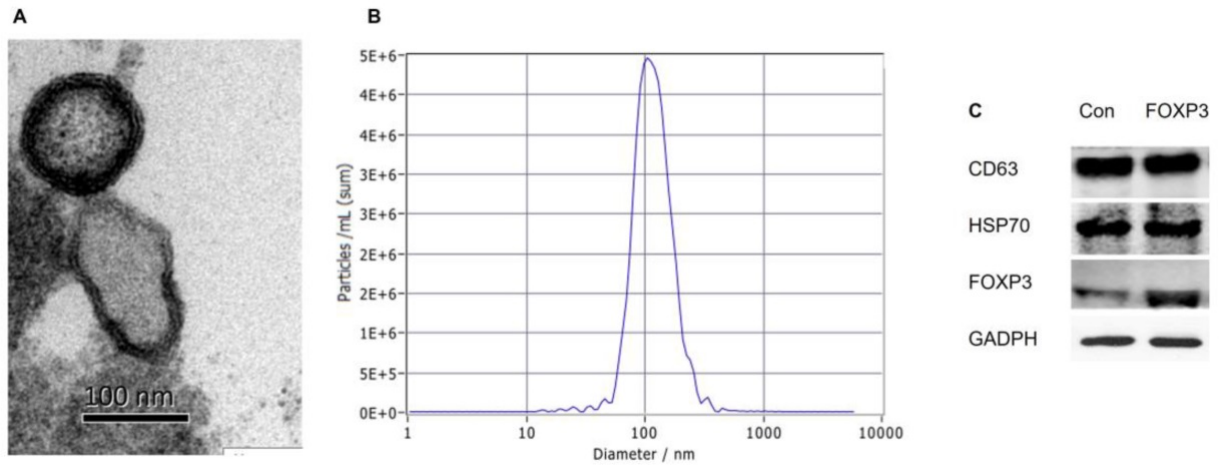
** Characterization of imDCs derived exosomes. (A)** EM analysis of exosomes from imDCs. Scale bar, 100 nm. **(B)** Size distribution profile analysis of exosomes. **(C)** Exosomes were analyzed by western blot for the presence of several proteins characteristic of exosomes as well as FOXP3. GADPH was used as the internal control.

The exosomes in the supernatants of cultured imDCs were isolated. EM observation revealed that both FOXP3-EXOs and Con-EXOs displayed oval-biconcave-shaped membranous vesicles (Fig. 2A). Further, the size distribution profile analysis was conducted by using the Particle Metrix ZetaView demonstrating a size peak of around 100nm of the exosomes (Fig. 2B). Additionally, exosomes derived from imDCs transfected with FOXP3 or not both had similar levels of exosome-associated HSP70 and CD63, while FOXP3-EXOs showed obviously elevated levels of FOXP3-EXOs than Con-EXOs (Fig. 2C).

### FOXP3-EXOs suppress T cell proliferation *in vitro*

**Figure 3:**
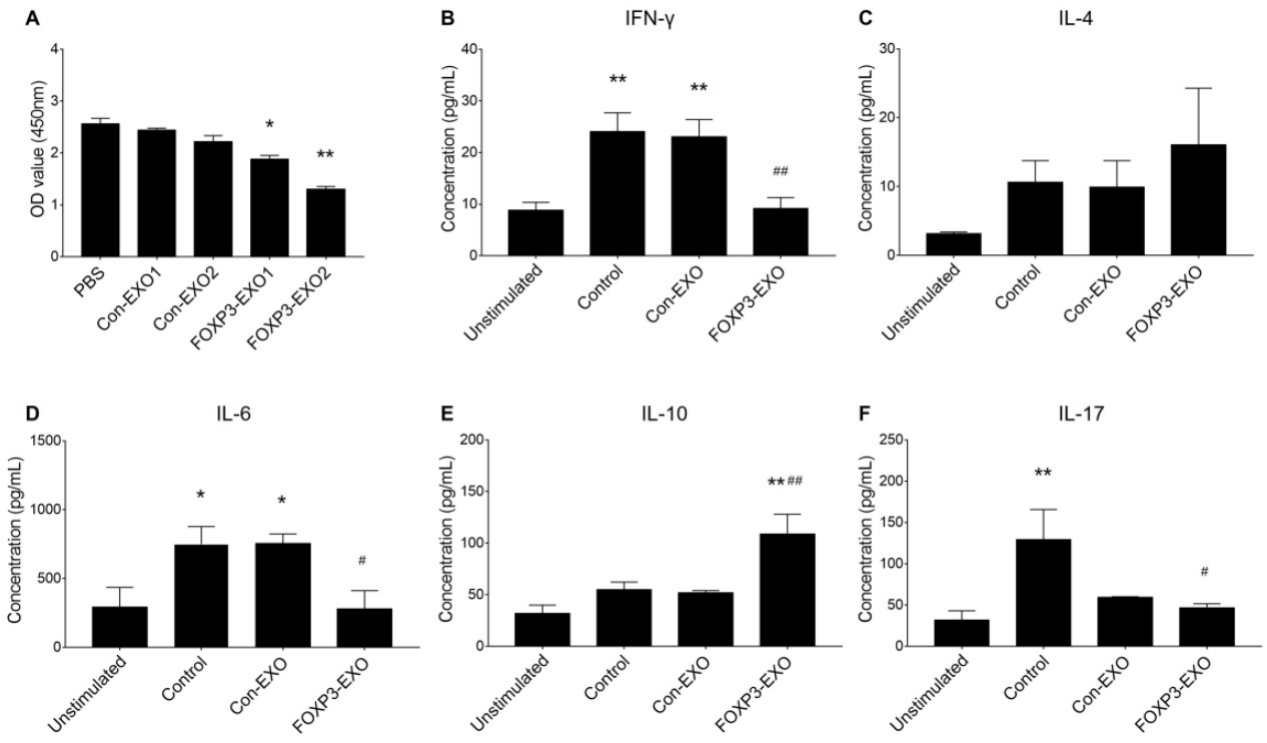
** The effects of FOXP3-EXOs on CD4^+^T cell proliferation and differentiation *in vitro*. (A)** FOXP3-EXOs significantly inhibited CD4^+^T cell proliferation in a dose-dependent manner assessed by CCK-8 assay. Con-EXO1 and FOXP3-EXO1 indicated 1ug/ml of exosomes of each type were used and Con-EXO2 and FOXP3-EXO2 indicated 2ug/ml of exosomes of each type were used for the assay. N=3, ^*^P < 0.05, ^**^P < 0.01 vs. the PBS group. **(B)** FOXP3-EXO obviously suppressed the production of INF-γ, IL-6, IL-17 and promoted the production of IL-10. Unstimulated group indicated cells were not activated by CD3/CD28 antibody beads, and groups of Control, Con-EXO, and FOXP3-EXO indicated cells were activated and treated with PBS, Con-EXOs, and FOXP3-EXOs, respectively. Data shown are expressed as the mean± SEM of each group (n=3). ^*^P < 0.05, ^**^P < 0.01 vs. the unstimulated group; ^#^P < 0.05, ^##^P < 0.01 vs. the control group.

To assess the ability of FOXP3-EXOs to inhibit T cell proliferation and differentiation, different EXOs were co-culture with CD4^+^T cell isolated from C57BL/6 mice. The results suggested that FOXP3-EXOs but not the Con-EXOs, significantly inhibited CD4^+^T cell proliferation in a dose-dependent manner (Fig. 3A). Based on the results above, 2ug/mL of exosomes (Con-EXOs, FOXP3-EXOs) were used to further evaluate the CD4^+^T cell differentiation. Treatment with FOXP3-EXOs, but not Con-EXOs significantly inhibited the secretion of INF-γ, IL-6, and IL-17 and promoted the production of IL-10 (Fig. 3B-F).

### Treatment with FOXP3-EXOs inhibits the development of EAE

**Figure 4:**
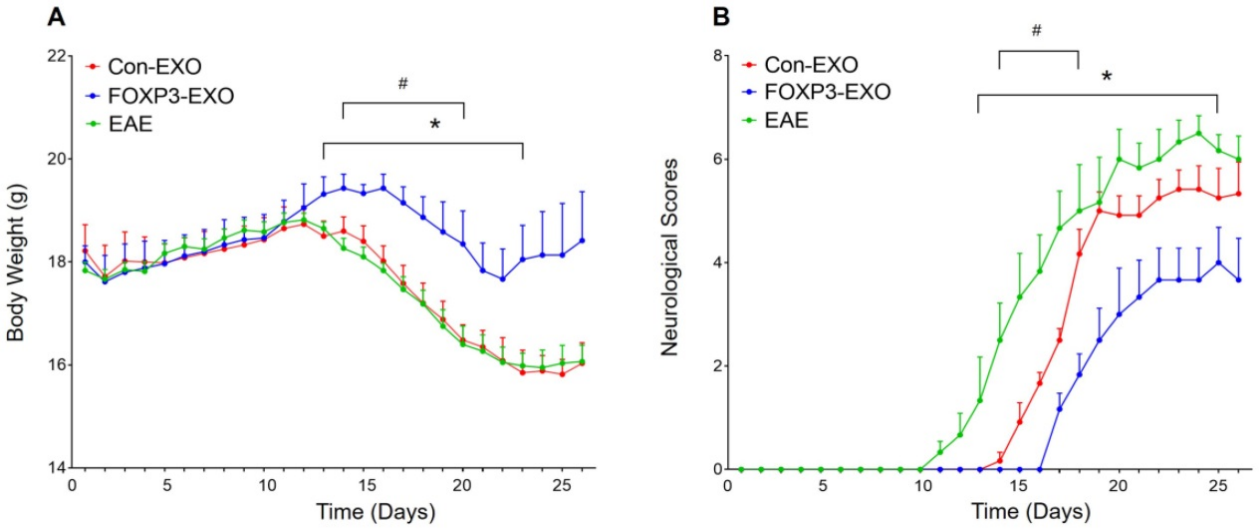
** FOXP3-EXOs ameliorate the loss of body weight and neuro-behavioral symptoms in EAE mice. (A)** Mice in FOXP3-EXOs group showed dramatically higher weights compared to those in the EAE and Con-EXOs groups. **(B)** FOXP3-EXOs treatment significantly slowed down the increase in clinical scores and reduced severity relative to the EAE mice with saline or Con-EXOs treatment. Control group indicated wildtype C57BL/6 mice treated with saline, and groups of EAE, Con-EXO, and FOXP3-EXO indicated EAE mice treated with saline, Con-EXOs, and FOXP3-EXOs, respectively. Data shown are expressed as the mean± SEM of each group (n=6). ^*^P < 0.05 vs. the EAE group; ^#^P < 0.05 vs. the Con-EXO group.

**Figure 5:**
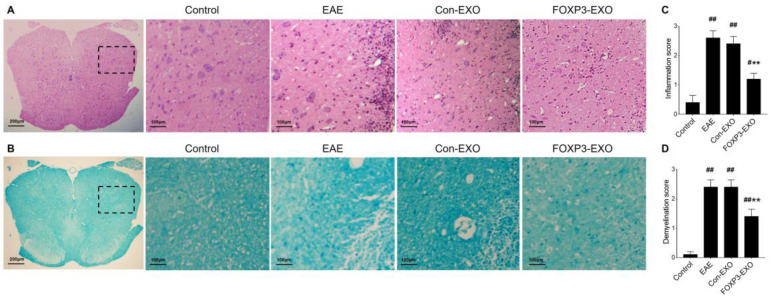
** FOXP3-EXOs treatment ameliorate inflammatory infiltration and demyelination in the spinal cord of EAE mice. (A)** High magnification of HE analysis of the spinal cord sections, Scale bar = 100 µm. **(B)** High magnification of LFB analysis of the spinal cord sections, Scale bar = 100 µm. **(C and D)** The mean scores of inflammation and demyelination in the EAE mice, Con-EXOs-treated mice and FOXP3-EXOs-treated mice. Data shown are representative images from each group or expressed as the mean±SEM of each group (n = 6). ^#^P < 0.05, ^##^P < 0.01 vs. the Control group;^ **^P < 0.01 vs. the EAE group.

**Figure 6:**
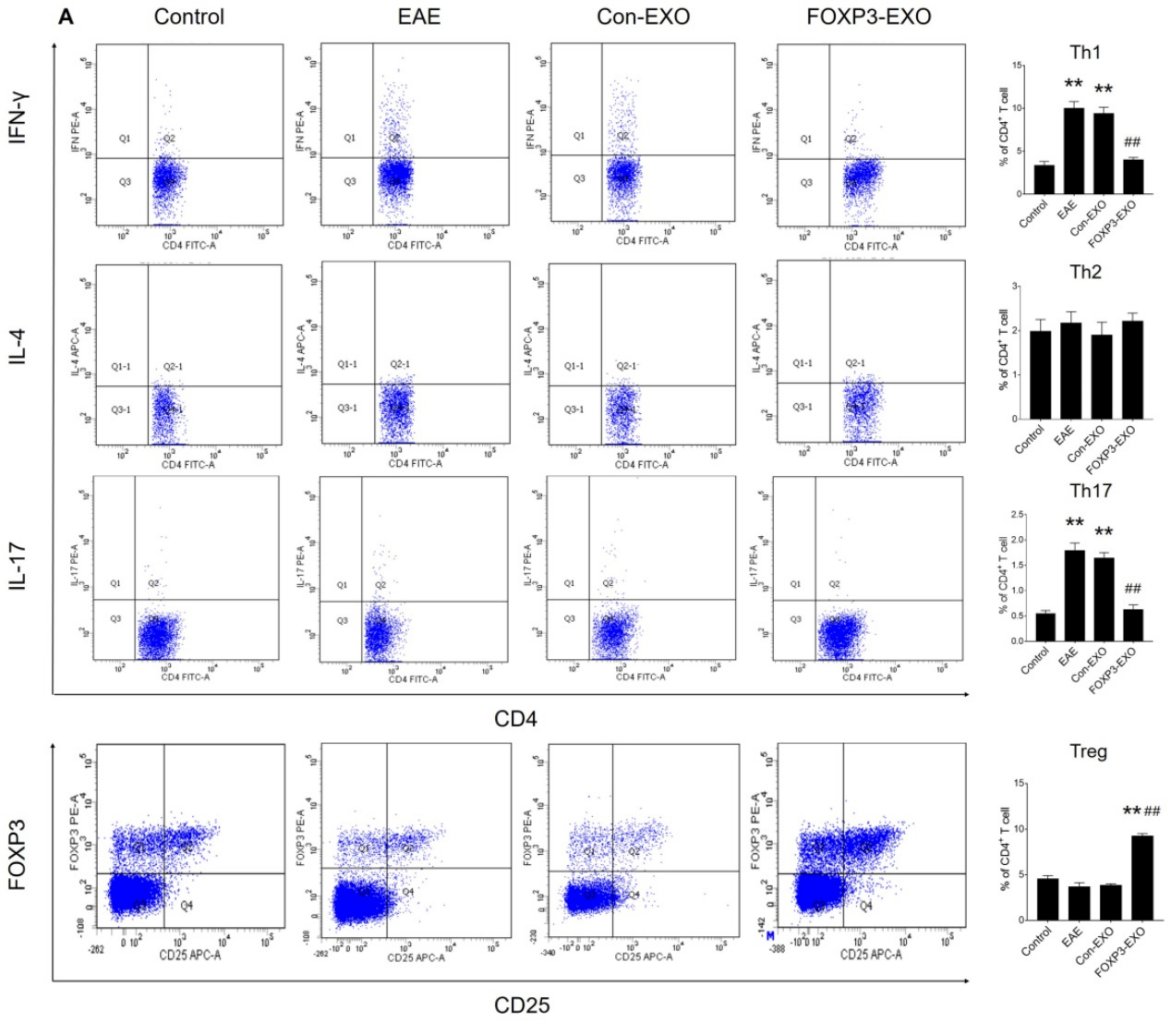

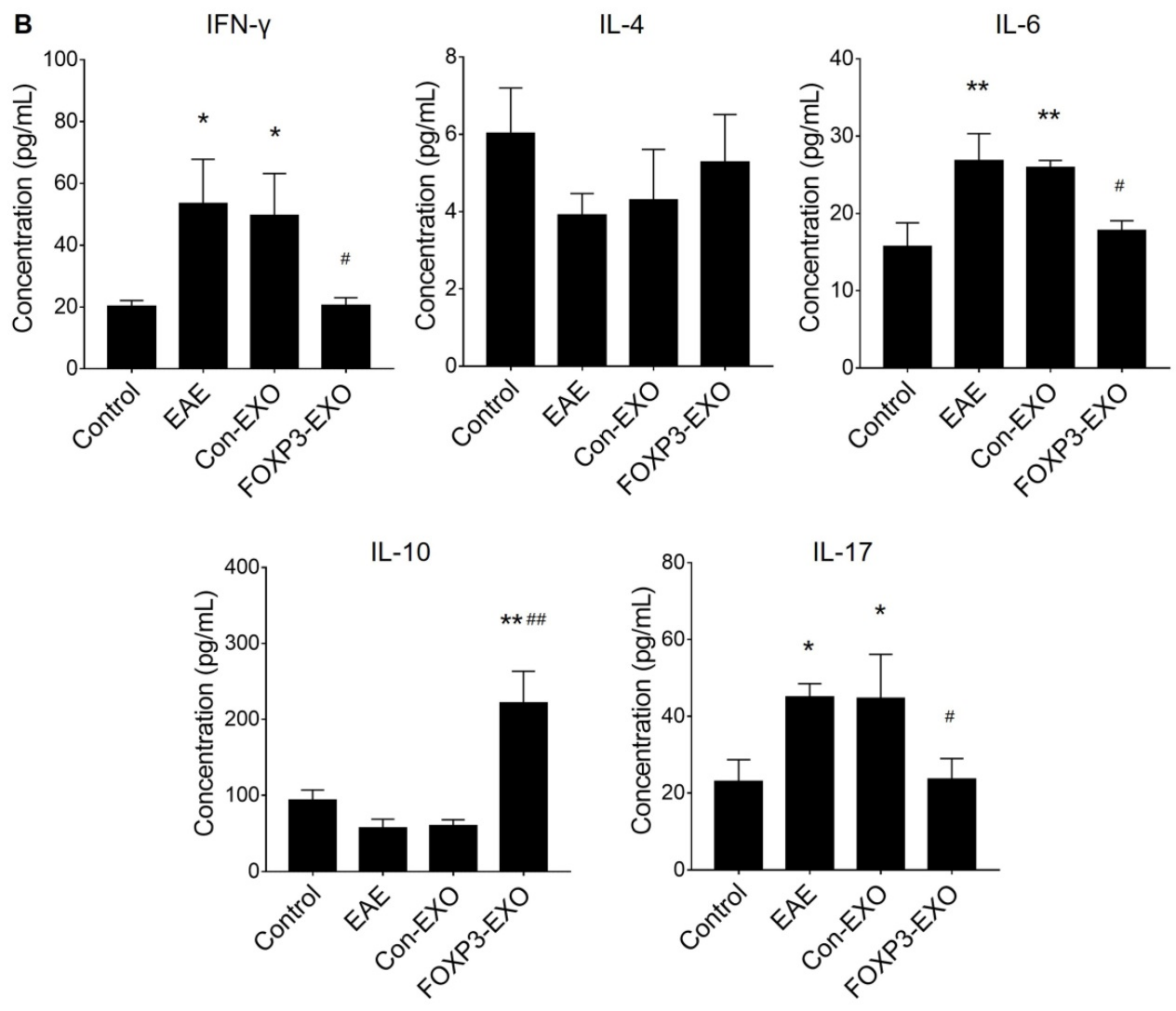
** FOXP3-EXOs treatment modulates immune responses in EAE mice. (A)** Flow cytometry analysis of the splenocytes culture of mice in each group (n=6 per group). **(B)** ELISA analysis of serum cytokines of mice in each group (n=6 per group). Data shown are representative images from each group or expressed as the mean± SEM of each group. ^*^ P < 0.05 or^ **^ P< 0.01 vs. the control group; ^#^ P < 0.05 or ^##^ P < 0.01 vs. the EAE group.

Given that Th/Treg imbalance is a key driver in the pathogenesis of EAE and the potential role of FOXP3-EXOs in inhibiting CD4^+^ T cell proliferation and reversing Th/Treg imbalance *in vitro*, we further tested whether treatment with FOXP3-EXOs could suppress the development of EAE. To this end, EAE was induced by MOG_35-55_ injecting to C57BL/6 mice, the mice were then randomized and treated with each type of exosomes on 3, 6, 9, 12, and 15 days postimmunization. Mice treated with FOXP3-EXOs showed dramatically higher weights compared to the EAE (from day 13 to day 23 post-immunization) and Con-EXOs (from day 14 to day 20 post-immunization) groups (Fig. 4A). Meanwhile, FOXP3-EXOs treated mice showed a significantly slower increase in clinical scores and reduced severity relative to the EAE (from day 13 to day 25 post-immunization) and Con-EXOs (from day 14 to day 18 post-immunization) groups (Fig. 4B). It is well-established that the typical pathology of EAE is characterized by inflammatory infiltration and demyelination of the CNS. Therefore, we aimed to further test the spinal cord pathological manifestations in EAE mice treated by FOXP3-EXOs with the goal of evaluating the influence of FOXP3-EXOs on the inflammatory infiltration and demyelination in the CNS of EAE model. The results demonstrated that FOXP3-EXOs could efficiently ameliorate the inflammatory infiltration and demyelination of EAE mice in the spinal cord, but these could not be observed in the Con-EXOs and saline treatment groups (Fig. 5). Alternatively, to preliminarily investigate the possible mechanisms underlying the action of FOXP3-EXOs, splenocytes isolated from the mice in each group were re-stimulated by MOG_35-55_ at day 25 post-immunization. The concentrations of Th1, Th2, Th17 and Treg cells were determined by flow cytometry. FOXP3-EXOs, but not Con-EXOs or saline treatment could significantly decrease the levels of Th1 and Th17 cells and increase the levels of Treg cells (Fig. 6A). Accordingly, treated with FOXP3-EXOs obviously reduced the levels of IFN-γ, IL-6 and IL-17 and raised the concentration of IL-10 in the serum of EAE mice (Fig. 6B). These demonstrations indicated that FOXP3-EXOs treatment could efficiently ameliorate the development of EAE by, at least partly, regulating the immune homeostasis of Th/Treg.

## Discussion

The present study preliminarily investigated the effects of FOXP3-EXOs on CD4^+^T cell proliferation and differentiation as well as on the development of EAE. The results demonstrated that DCs-derived FOXP3-EXOs could significantly inhibit the CD4^+^T cell proliferation, reduced the concentrations of Th1, Th17 cells and related cytokines (IFN-γ, IL-6 and IL-17), and promote the production of Treg cells and IL-10. However, FOXP3-EXOs treatment did not affect the levels of Th2 cells and IL-4 both *in vitro* and *in vivo*. In addition, treatment with FOXP3-EXOs efficiently inhibited the development of EAE and ameliorated the inflammatory infiltration and demyelination in the spinal cord, the typical pathological manifestations of EAE. Taken together, FOXP3-EXOs exhibited favorable immunomodulation and induction of immune tolerance *in vitro* and *in vivo*, which made FOXP3-EXOs a promising candidate for MS treatment.

It is well-established that Th1 and Th17 cells not only represented a major initiator but was also a participant in the pathogenesis and progression of MS as well as its animal model—EAE: 1) In MS, raised proportion of Th1 and Th17 cells in the peripheral blood and increased concentrations of Th1- and Th17-related cytokines such us IFN-γ, TNF-α, IL-6, IL-17, IL-21, IL-22, and IL-23 in the serum had been mentioned in numerous previous studies 2, 9, 29. 2) MOG-specific- Th1 and Th17 cells could induce typical adoptive transfer EAE 30. In contrast, Treg cells secreting the anti-inflammatory cytokine such as IL-10, had been reported to inhibit the production of pro-inflammatory cytokines and induce the immunosuppression, which guaranteed their potential contribution in maintaining the immune homeostasis in a variety of physiological and pathological settings 31, 32. Accordingly, accumulating lines of evidences had indicated that it is the imbalance of Th/Treg that causes the dysregulation of inflammatory and anti-inflammatory factors in the body ultimately leads to the development of MS 33, 34. Therefore, regulation of Th/Treg imbalance through multiple modalities is expected to be a promising strategy for the treatment of MS.

It is well-established that the activation, proliferation, and differentiation of CD4^+^T cells depends on antigen-presenting cells, and DCs was known to be the most crucial antigen-presenting cell in the body. The role of DCs in T cell-mediated immunity and tolerance is dependent on their maturation status, and thus T cell responses can be indirectly mediated by manipulating the maturation phenotype of DCs 11.

Notably, our previous study had demonstrated that imDCs transfected with FOXP3 could apparently inhibit the production of Th1 and Th17 cells and their related cytokines and promote the responses of Treg cells *in vitro* 17. FOXP3 is known to be required for the development and function of naturally occurring Treg cells, and its expression is sufficient to convert nonregulatory CD4^+^CD25^-^ T cells into T cells with regulatory activity 35. As mentioned above, FOXP3-expressing Tregs are important for the maintenance of peripheral tolerance and perturbations in the development of Tregs is known to be associated with autoimmune diseases such as MS. These findings drive us to hypothesize that imDCs transfected with FOXP3 may be able to suppress the EAE development by regulating the Th/Treg balance. However, direct application of DCs for immunotherapy has significant limitations: 1) limited DCs proliferation, which may not meet the requirements of clinical therapy; 2) live cell vaccine is complicated in quality control, weak in production and storage, and high in cost.

Exosomes, a recently discovered subtype of membrane vesicle released from the cell and presumably reflect the function of the cells at the time of isolation may serve as a therapeutic alternative to DCs 19. In the present study, the FOXP3-EXOs isolated from imDCs were capable to inhibit the production of Th1, Th17 cells and promote the production of Treg cells *in vitro* and *in vivo*. These findings were somewhat different from those in our previous study. In that study, imDCs transfected with FOXP3 could significantly suppress the production of Th1 and Th17 and promote the production of Th2 and Treg. We considered that the failure of FOXP3-EXOs to inhibit Th2 cell production may be due to potentially different action modes and mechanisms of exosomes and imDCs in regulating the CD4^+^ T cell differentiation. The activation and differentiation of CD4^+^ T cells require interaction with MHC class II and co-stimulatory molecules expressing on the surface of DCs. While, imDCs show low surface expression of costimulatory molecules, have only modest levels of MHC class II, and comparatively high surface expression of co-inhibitory molecules such as PD-L1 and CD80/CD86. imDCs-derived exosomes: 1) carry the surface molecules of imDCs and regulate CD4^+^ T cell activation and differentiation in a way similar to imDCs; 2) carry DNA and RNA (MicroRNA, lncRNA, CircRNA) molecules from imDCs that enter CD4^+^ T cells through fusion with cell membranes, receptor-ligand interactions, and endocytosis, and then interact with DNA or RNA and mediate related gene expression to regulate cell activation and differentiation. Therefore, further studies were necessarily needed to elucidate the underlying mechanisms. Additionally, FOXP3-EXOs could efficiently ameliorate the development of EAE and inhibit the inflammatory infiltration and demyelination in the CNS. Considering the pro-inflammatory role of Th1 and Th17 cells and the anti-inflammatory role of Treg cells in MS pathogenesis, and our findings that FOXP3-EXOs regulated Th/Treg immune homeostasis *in vitro* and *in vivo*, we suggest that the suppression of EAE development by FOXP3-EXOs may be related to its reversion of Th/Treg immune imbalance.

Several limitations were needed to be mentioned. Firstly, the present study focused on the spinal cord for the pathological assessment, and the corresponding evaluations in the brain were lacking. Secondly, the relevant detection of the transcription factors of Th and Treg cells were lacking. Thirdly, there was no further investigation regarding the detailed mechanism of Th/Treg regulated by FOXP3-EXOs *in vitro* and *in vivo*. Fourthly, the investigations of mechanisms underlying the different actions of imDCs transfected with FOXP3 and FOXP3-EXOs on Th2 cell were not conducted in the present study. Therefore, further studies investigating the potential mechanisms of FOXP3-EXO in regulating Th/Treg homeostasis *in vitro* and *in vivo* will necessarily need to be conducted in near future.

## Conclusions

The present study preliminarily investigated the effects and potential mechanisms of exosomes from DCs transfected with FOXP3 in EAE and revealed that the FOXP3-EXOs could inhibit the production of Th1 and Th17 cells and promote the production of Treg cells *in vitro* and *in vivo* as well as ameliorate the development of EAE. The neuroprotective effects of FOXP3-EXOs on EAE are likely due to the regulation of Th/Treg balance.
